# Epigenomic Alterations in Breast Carcinoma from Primary Tumor to Locoregional Recurrences

**DOI:** 10.1371/journal.pone.0103986

**Published:** 2014-08-06

**Authors:** Matahi Moarii, Alice Pinheiro, Brigitte Sigal-Zafrani, Alain Fourquet, Martial Caly, Nicolas Servant, Véronique Stoven, Jean-Philippe Vert, Fabien Reyal

**Affiliations:** 1 Centre for Computational Biology, Mines ParisTech, Fontainebleau, France; 2 Institut Curie, Paris, France; 3 U900, INSERM, Paris, France; 4 UMR144, Oncology Molecular Team, Institut Curie, Paris, France; 5 Department of translational research, Residual Tumor and Response to Treatment Team, Institut Curie, Paris, France; 6 Department of Tumor Biology, Institut Curie, Paris, France; 7 Department of Radiotherapy, Institut Curie, Paris, France; 8 Department of Surgery, Institut Curie, Paris, France; CEA - Institut de Genomique, France

## Abstract

**Introduction:**

Epigenetic modifications such as aberrant DNA methylation has long been associated with tumorogenesis. Little is known, however, about how these modifications appear in cancer progression. Comparing the methylome of breast carcinomas and locoregional evolutions could shed light on this process.

**Methods:**

The methylome profiles of 48 primary breast carcinomas (PT) and their matched axillary metastases (PT/AM pairs, 20 cases), local recurrences (PT/LR pairs, 17 cases) or contralateral breast carcinomas (PT/CL pairs, 11 cases) were analyzed. Univariate and multivariate analyzes were performed to determine differentially methylated probes (DMPs), and a similarity score was defined to compare methylation profiles. Correlation with copy-number based score was calculated and metastatic-free survival was compared between methods.

**Results:**

49 DMPs were found for the PT/AM set, but none for the others (FDR 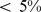). Hierarchical clustering clustered 75% of the PT/AM, 47% of the PT/LR, and none of the PT/CL pairs together. A methylation-based score (MS) was defined as a clonality measure. The PT/AM set contained a high proportion of clonal pairs while PT/LR pairs were evenly split between high and low MS score, suggesting two groups: true recurrences (TR) and new primary tumors (NP). CL were classified as new tumors. MS score was significantly correlated with copy-number based scores. There was no significant difference between the metastatic-free survival of groups of patients based on different classifications.

**Conclusion:**

Epigenomic alterations are well suited to study clonality and track cancer progression. Methylation-based classification of TR and NP performed as well as clinical and copy-number based methods suggesting that these phenomenons are tightly linked.

## Introduction

Breast conservative therapy, consisting in a partial mastectomy followed by whole breast irradiation, is the standard treatment for patients with early stage breast cancer. Overall survival is not significantly different from more physically and psychologically aggressive treatments such as mastectomy [1]. However, patients relapse within 10 years in the same breast as the primary tumor (PT) in approximately 6 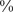 of cases [2], and within 5 years in the contralateral breast in approximately 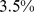 of cases [3] or more in BRCA1/2 mutation carriers [4]. Moreover, at the time of diagnosis, early stage breast cancers have already spread to axillary lymph nodes in roughly 30 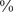 of cases [5].

These different types of locoregional evolutions have different implications in terms of survival and treatments. Axillary metastases (AM) is usually predictive of poor survival [6] and is considerably worsen in triple negative breast cancers [7]. Local recurrences (LR) have been tightly linked with a greater risk of distant metastasis [8]. Veronesi et al. [9] distinguished two categories of local recurrences: true recurrences (TR), corresponding to re-growth of resistant cells after initial treatment, and new primary tumors (NP), corresponding to *de novo* cancer. This classification is of potential interest to define adapted treatment scheme, as NP are considered to have an improved survival compared to TR [10]. Contralateral breast cancers (CL) are also an heterogeneous entity depending on the synchronism with the primary tumor. Synchronous bilateral breast cancers are developed at the same time, with the same genetic, environmental and hormonal background as the PT. Metachronous CL are usually treated as new cancers [11] although a rare portion are considered as metastases. Overall, CL are still associated with a greater risk of metastasis compared to patients without CL [12].

Differences between the PT and either the AM, the LR or the CL have been studied at the genomic, transcriptomic and proteomic levels. Ellsworth et al. [13] showed an overall frequency of allelic imbalance greater in PT than in AM. Weigelt et al. [14] explored the gene expression profile of PT and their matched AM but were not able to identify a subset of genes to discriminate them, while Feng et al. [15] identified a set of 79 genes able to differentiate PT from matched AM. Studies between PT and LR have mainly focused on distinguishing TR and NP. A criterion based on clinical and pathological features was first established but judged insufficiently robust for most clinical applications. Several studies investigated the difference between TR and NP based on pangenomic analyzes of DNA copy number alterations (CNA) [16], [17], intratumoral immune responses [18], loss of heterozigosity [19], to p53 analysis [20], or X-chromosome inactivation [21]. Finally, studies of PT and CL highlighted the role of synchronism of the CL. Similarity measures based on DNA copy number profiles [22] or allelic imbalance [23] showed a higher level of similarity between PT and synchronous CL compared to PT and metachronous CL.

Epigenetic modifications in cancer has recently been the topic of many studies. In particular the link between hypermethylation and gene silencing is well known [24]–[26]. Several studies have then focused to describe cancer as an epigenetic disease. Baylin et al. [27] have shown that aberrant hypermethylation of specific regions, dominantly CpG islands, are linked with the silencing of tumor suppressor genes and that this phenomenon is present in most cancers. Laird [28], Ehrlich [29] and Das [30] suggested that a global hypomethylation phenomenon was also linked with tumorogenesis. Jones [31] made a complete review of the hallmarks of epigenomics associated with cancer. Moreover, DNA methylation is conserved during cell division [26], [32] and could serve as a measure for clonality between cells in the classification of LR as either TR or NP.

In this study, epigenetic differences as well as similarities between PTs and either their AMs, LRs or CLs are analyzed. In the first part, univariate and multivariate analyzes are performed between the methylome profiles of primary tumors and their matched recurrences to observe recurrent patterns in cancer progression. Then in the second part, epigenome-wide similarity analyzes on the same samples is performed to observe clonality between tumor cells.

## Results

### Methylation differences between PT and their matched metastasis or recurrence

A collection of 17 PT/LR pairs, 11 PT/CL pairs, and 20 PT/AM pairs was analyzed. The methylation data are available in the GEO database record number: GSE44870. Tables 1, 2 and 3 detail the summarized clinico-histopathological properties of each sample. Some of the PT/LR samples match in part the cohort studied by Bollet et al. [16], and the corresponding sample numbers from both studies are provided in Table 2. Tables S1, S2 and S3 provide more detailed characteristics.

**Table 1:** Summarized PT/LR Clinical and histological features.

			PT					Local Recurrence						Discordance
Pair	Cor	Age	Type	Grade	ER	PR	HER2	Type	Grade	ER	PR	HER2	Loc	
1	1	23.3	D	3	-	+	-	D	2	+	+	-	1	ER
2	3	42.9	D	3	+	+	-	D	3	+	+	-	1	
3	11	49.3	L	3	-	-	-	D	3	-	-	+	1	HER2
4	16	48.8	D	2	+	+	-	D	1	+	+	-	1	
5	12	49.3	L	2	+	+	-	L	2	+	-	-	0	PR
6	13	45.4	D	2	+	+	-	D	2	+	+	-	1	
7	15	46.5	D	2	+	+	-	D	2	+	+	-	1	
8	2	42.4	D	2	+	+	-	L	1	+	+	NA	1	
9	4	48.6	L	1	+	+	-	L	2	+	+	-	1	
10	14	44	L	2	+	+	-	L	2	-	+	-	1	ER
11	18	NA	D	3	-	-	NA	D	2	+	+	NA	1	ER/PR
12	20	47.5	D	3	-	-	+	D	3	-	-	+	0	
13	21	46.7	D	2	+	-	NA	D	3	+	-	-	1	
14	23	31	D	2	-	-	-	D	3	-	-	-	1	
15	24	48.1	D	3	-	-	-	D	3	-	-	-	1	
16	25	43.3	D	3	+	+	-	D	3	+	+	-	1	
17	26	30.8	D	3	-	-	-	D	3	-	-	-	1	

**Cor** (Correspondence): correspondence number with the Bollet/Servant cohort from [16], **Type**: histological type of the tumor (D =  ductal, L =  lobular), **Grade**: Aggressiveness of the tumor (1 to 3), **ER**: presence of estrogen receptors, **PR**: presence of progesterone receptors, **HER2**: presence of HER2 receptors, **Loc** (Location): 1 if the recurrence was located less than 4cm from the PT.

**Table 2:** Summarized PT/CL Clinical and histological features.

		PT					Contralateral Recurrence					Discordance
Pair	Age	Type	Grade	ER	PR	HER2	Type	Grade	ER	PR	HER2	
1	46.6	L	3	+	+	-	NA	NA	+	+	-	
2	46.9	D	2	+	+	-	D	2	+	+	-	
3	48.4	D	3	+	+	-	D	3	+	+	-	
4	42.6	D	2	-	-	-	D-L	2	+	+	-	ER/PR
5	48.5	D	2	+	+	-	D	3	+	+	-	
6	44.5	D	2	+	+	-	Med	2	-	-	-	ER/PR
7	46	D	2	+	+	-	D	1	+	+	-	
8	48.9	D	3	+	+	-	Meta	3	-	-	-	ER/PR
9	38.9	D	3	-	-	-	D	3	+	+	-	ER/PR
10	31	D	3	-	-	-	D	3	-	-	-	

**Type**: histological type of the tumor (D =  ductal, L =  lobular, Med = Medullary, Meta = Metaplasic), **Grade**: Aggressiveness of the tumor (1 to 3), **ER**: presence of estrogen receptors, **PR**: presence of progesterone receptors, **HER2**: presence of HER2 receptors.

**Table 3:** Summarized PT/AM Clinical and histological features.

Pair	Age	Type	Grade	ER	PR	HER2
1	45.9	D	3	+	+	-
2	NA	D	3	+	+	-
3	NA	NA	NA	+	+	-
4	48.8	D	1	+	+	-
5	43.6	D	3	-	-	-
6	35.3	D	2	+	+	-
7	45.1	D	3	+	+	-
8	41.9	D	2	+	+	NA
9	43.5	D	1	+	+	-
10	43.7	D	3	+	+	NA
11	44.9	D	2	-	-	-
12	43.6	D	1	+	-	-
13	40.2	D	3	-	-	+
14	32.5	L	3	+	+	+
15	38.5	D	2	-	+	-
16	37.5	D	3	+	+	-
17	39.3	D	3	+	+	-
18	37.6	D	3	-	-	-
19	36.6	D	3	+	+	+
20	35.4	D	3	-	+	-

**Age**: Age of the patient at diagnosis of the primary tumor in years, **Type**: histological type of the tumor (D =  ductal, L =  lobular, Meta = Metaplasia), **Grade**: Aggressiveness of the tumor (1 to 3), **ER**: presence of estrogen receptors, **PR**: presence of progesterone receptors, **HER2**: presence of HER2 receptors.

Within each of the three cohorts, pairs of tumors including a PT and a metastatic or relapse sample can be used to investigate whether particular patterns in methylation profiles can serve as marker for cancer progression.

Within each cohort, investigations were made to detect differences at the methylome level between PT and the corresponding matched metastasis (AM) or relapse samples (LR or CL). Using a paired Wilcoxon test, 49 probes significantly differentially methylated were found between PT and AM samples (at a 5% FDR level). The top 50 probes ranked by p-value and the corresponding genes are listed in Table 4. This suggests that a general signal characteristic of cancer progression from PT to AM might exist. However, no probe was found significantly differentially methylated between PT and LR, and between PT and CL. This may be due to the lack of cancer progression marker at the methylation level between PT and relapse, to the fact that most relapses may not be biologically related to the PT, or to the small size of the cohort which limits the power of statistical tests. The top 50 probes ranked by p-value then by absolute methylation variation between the primary tumor and its recurrence is also provided in Tables S4 (PTLR) and S5 (PTCL). No overlap existed between the three lists except for one gene (*PI3K5R* between the PT/AM and PT/LR datasets). All the corresponding quantile-quantile plots are available in Figure S1.

**Table 4:** Significantly differentially methylated genes between PT and AM samples.

CpG	Gene	Pvalue	Methylation Variation
cg20161089	*IFI27*	0.013	0.238
cg18140857	*RDHE2*	0.013	0.102
cg04619381	*LOC222171*	0.013	−0.048
cg23698969	*SLC22A18*	0.013	0.042
cg16179125	*CTSZ*	0.020	0.182
cg24959428	*GBP6*	0.020	0.126
cg22630748	*INHBE*	0.020	0.100
cg03623878	*MCF2L*	0.020	−0.050
cg25115460	*TP73*	0.022	0.109
cg11946165	*CTSK*	0.022	0.098
cg01318557	*LAT2*	0.022	0.063
cg13453139	*PIK3R5*	0.022	−0.063
cg21416237	*FKBP10*	0.028	0.085
cg19814116	*KCNAB2*	0.031	−0.217
cg22392666	*FXYD7*	0.031	−0.217
cg18212039	*EXTL1*	0.031	0.106
cg03532879	*SMAF1*	0.031	0.041
cg27149093	*SLC41A2*	0.032	0.145
cg18946226	*MYR8*	0.032	0.139
cg15448245	*GGTLA1*	0.032	0.132
cg15792367	*KLK11*	0.032	0.111
cg07459489	*SLC30A8*	0.032	0.085
cg15021292	*PIK3R1*	0.032	0.075
cg26267561	*OXT*	0.032	0.072
cg08647446	*RASSF6*	0.032	−0.070
cg20967028	*ART4*	0.032	−0.062
cg08550724	*C6orf182*	0.032	0.052
cg09737668	*SLAMF9*	0.032	0.049
cg23036025	*SLC27A5*	0.032	0.047
cg15296858	*PPM1G*	0.032	0.038
cg04961553	*OCIAD2*	0.032	−0.018
cg17558126	*RASSF5*	0.038	−0.137
cg06852652	*CYP2C18*	0.038	0.055
cg05840031	*PAX6*	0.038	−0.031
cg15043801	*DNMT1*	0.038	−0.016
cg05649009	*CHRNA1*	0.046	0.171
cg16176379	*AYTL1*	0.046	−0.128
cg05538432	*C1S*	0.046	0.123
cg25151295	*RANBP5*	0.046	0.121
cg23841186	*SOAT2*	0.046	0.096
cg05656364	*VAMP8*	0.046	−0.085
cg14833385	*HLA-DMA*	0.046	−0.085
cg27655855	*CST9L*	0.046	0.084
cg27461196	*FXYD1*	0.046	0.069
cg14106308	*VEPH1*	0.046	−0.056
cg22857604	*RASSF5*	0.046	−0.053
cg10891879	*CASZ1*	0.046	−0.029
cg25042226	*PAX8*	0.046	−0.025
cg11655418	*RPS10*	0.046	0.005

**CpG**: CpG probe name. **Gene**: Associated gene. **Pvalue**: FDR corrected p-value. **Methylation Variation**: Mean variation of methylation from the primary tumor to the axillary metastasis.

**Figure 1:**
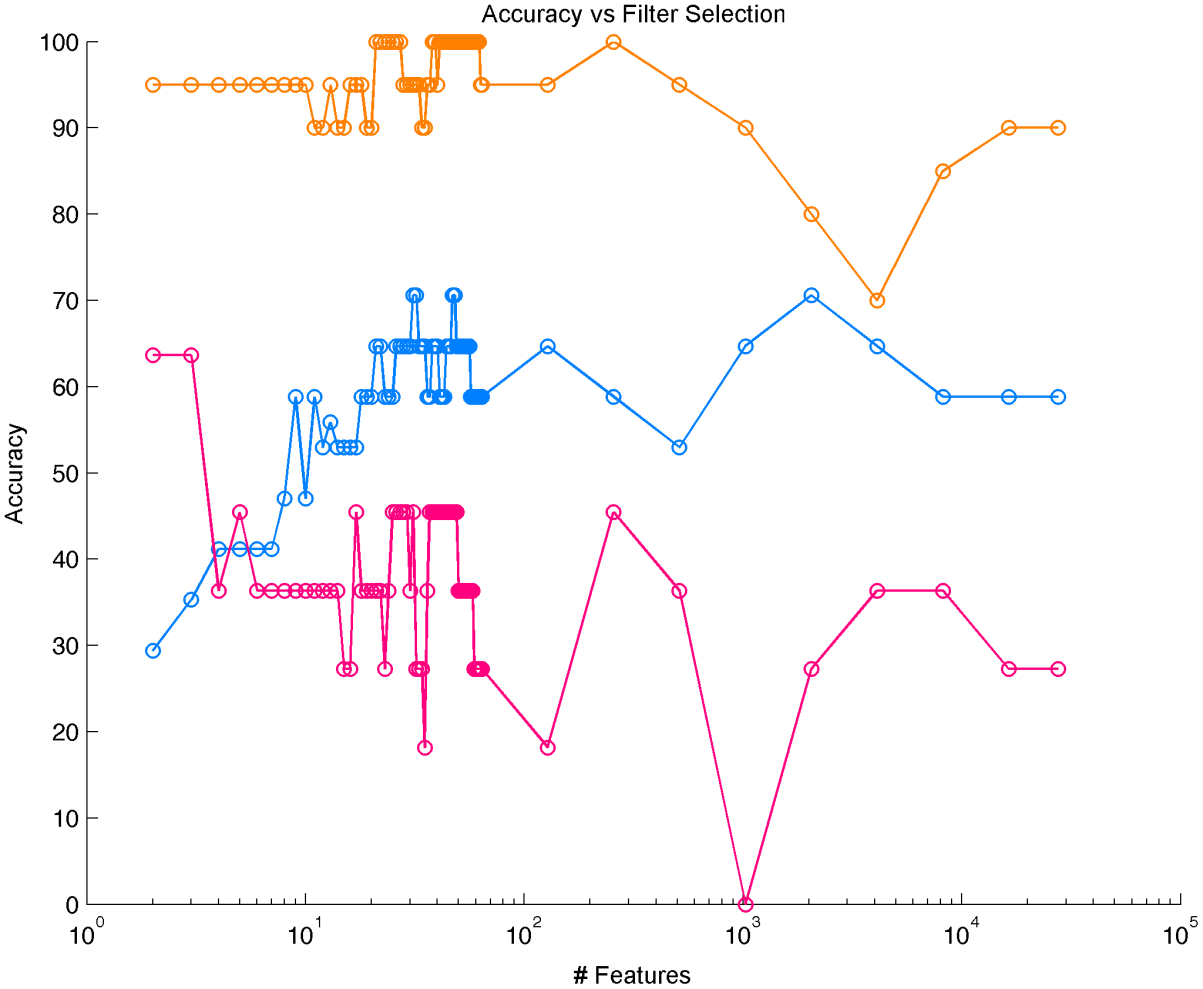
Accuracy of multivariate analysis with respect to feature selection to classify primary tumors from locoregional evolutions. Accuracy to classify PT from AM (resp. LR, resp. CL.) is represented in yellow (resp. blue, resp. pink).

On the PT/AM cohort, the SVM model correctly identified the PT and AM in 18 out of 20 held-out pairs (90% success rate, P-value =  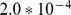) when considering the whole methylation profile probes. The SVM model obtained after dimensionality reduction by filtering the 22 most significant probes selected according to a Wilcoxon test gave a 100% accuracy. As illustrated in Figure 1, good accuracy was still achieved when considering an increasing number of probes (Accuracy 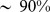). On the PT/LR and PT/CL cohorts, however, the success rate was respectively 58% (10 out of 17 pairs, P-value = 0.31) and 27% (3 out of 11 pairs, P-value = 0.11) when taking all probes into account. Note that these values are not significantly different from random guess.

### Methylation conservation between PT and their matched metastasis or recurrence

**Figure 2:**
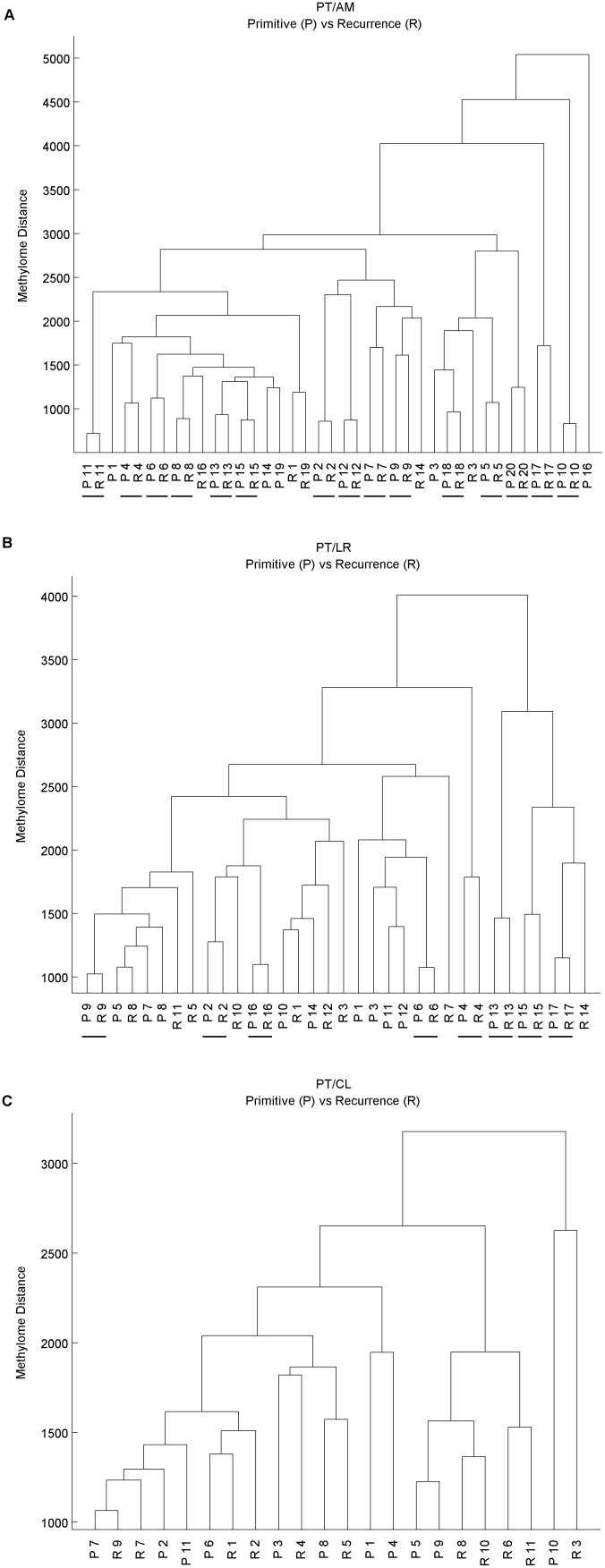
Study of similarity between matched primary tumors and recurrences by hierarchical clustering. Hierarchical clustering based on the manhattan distance between methylome profiles with complete linkage was performed. Real pairs that are closer to each other than to any other samples are underlined. Panel A (resp. B, resp. C) represents the PT/AM (resp. PT/LR, resp. PT/CL) set.

Instead of searching for differences between PT and their matched metastasis or recurrence, which may characterize markers for cancer progression, the study also focuses on similarities between methylation profiles, which may be useful for example to characterize clonality between a PT and a recurrence. A hierarchical clustering was first performed for all samples within each cohort to characterize the similarities between real matched pairs compared to unrelated samples. The resulting dendrograms are presented in Figure 2. Interestingly we see that matched pairs of PT and metastasis/recurrence samples are usually closer to each other than to any unrelated tissues in the PT/AM cohort (15 out of 20, 75%), less often in the PT/LR cohort (8 out of 17, 41%), and never in the PT/CL cohort. This observation is consistent with decreasing proportions of real clonal pairs from the PT/AM to the PT/CL set.

**Figure 3:**
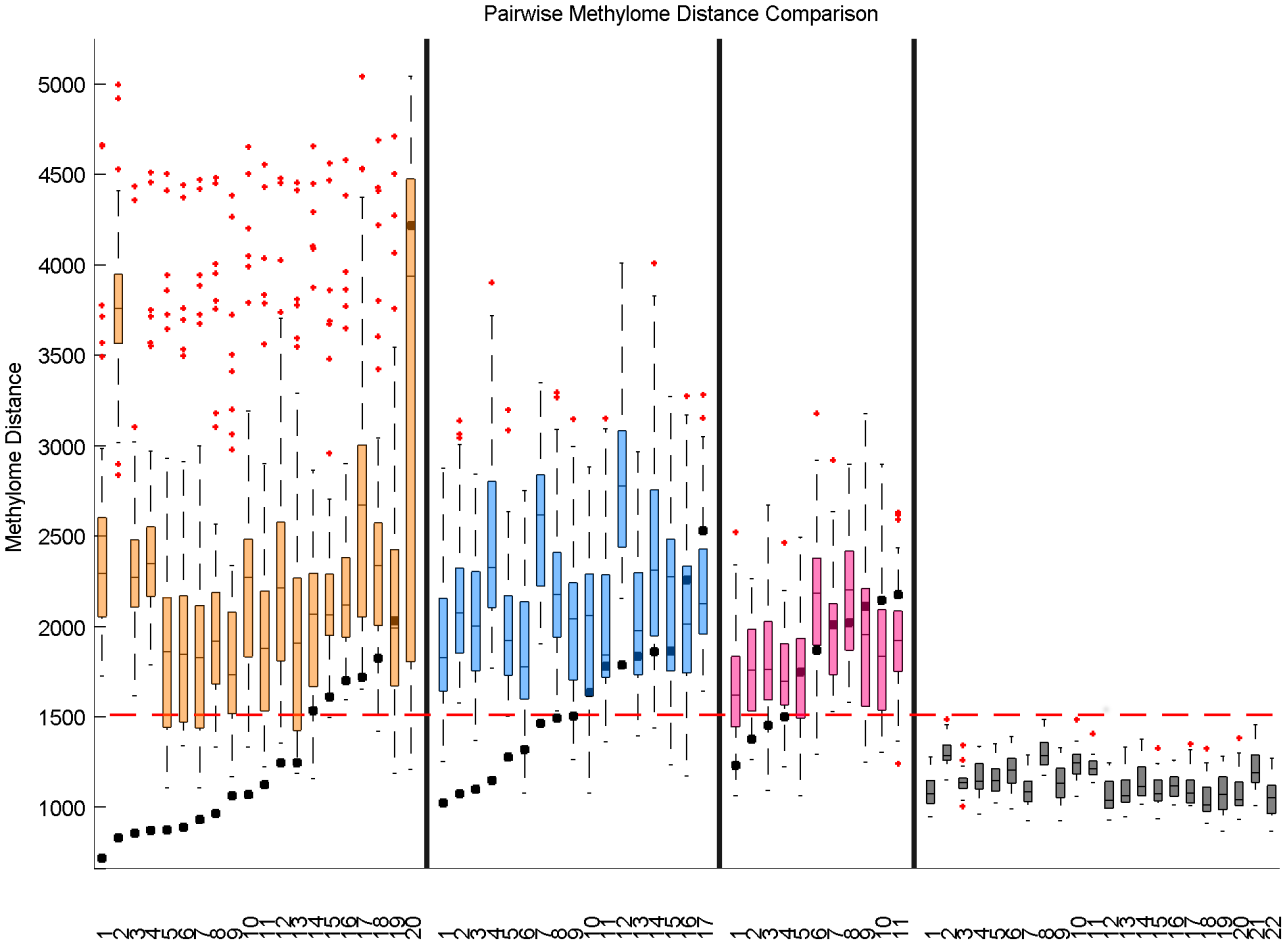
Distribution of methylation distances between different samples pairs for each groups. **Real**: boxplot of methylome distances for all matched pairs that is a PT and its corresponding metastasis or recurrence. **Artificial**: boxplot of methylome distances for all unmatched pairs that is a PT and an unrelated metastasis or recurrence. **Primary**: boxplot of methylome distances to distances between two PT of two different individuals. **Recurrence**: boxplot of methylome distances between two metastasis or recurrence samples of two different individuals.

**Figure 4:**
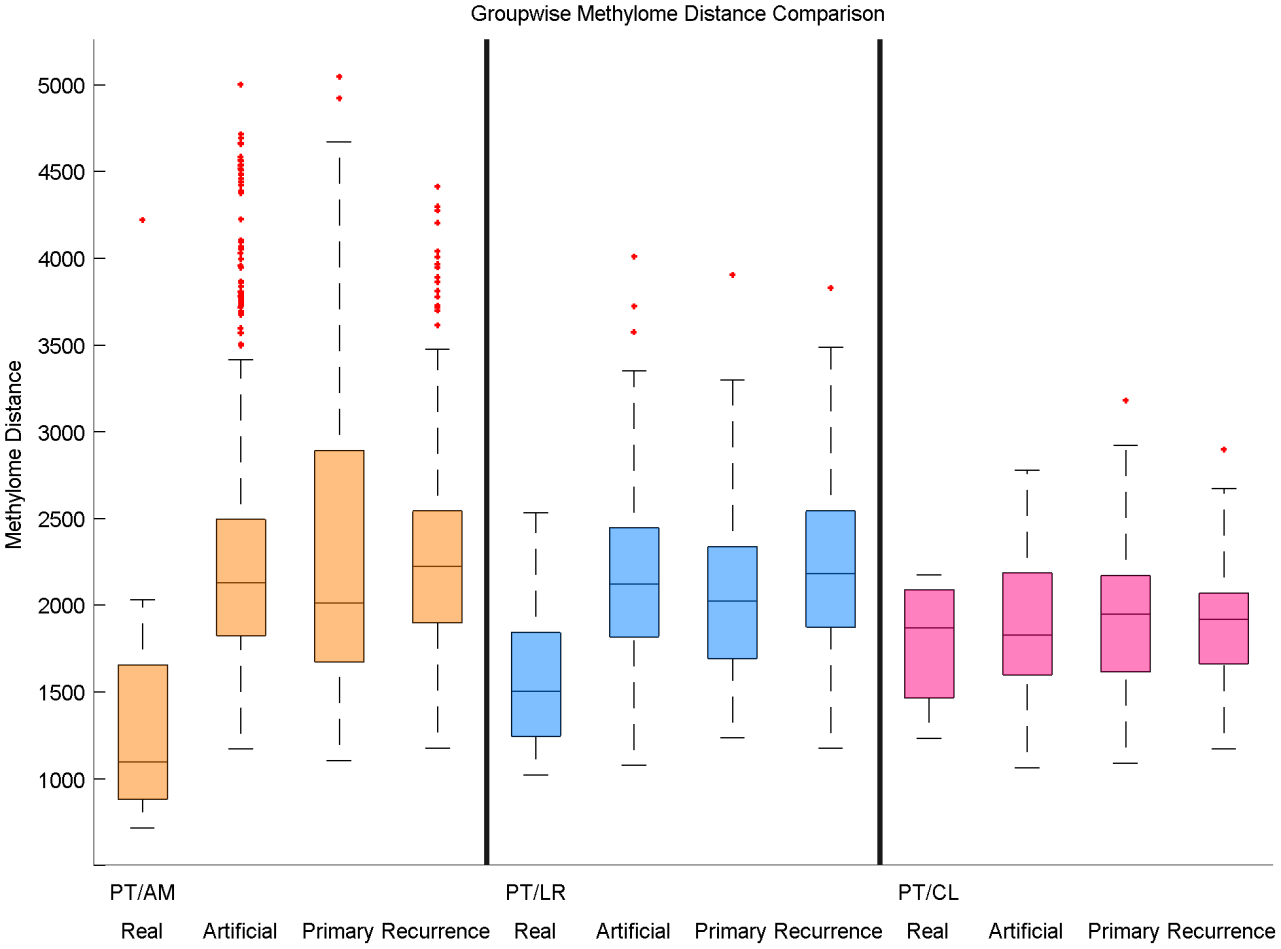
Pairwise methylome distance for each samples. Each boxplot represents the Manhattan distance between primary tumor 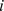 and an unrelated locoregional evolution, or the Manhattan distance between locoregional evolution i and an unrelated primary tumor. The black square represent the Manhattan distance between the matched primary tumor and locoregional evolution from sample 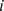. The yellow (resp. blue, resp. pink) panel represents the PT/AM (resp. PT/LR, resp. PT/CL) set. The last panel represents the distribution of distances between the healthy breast tissue 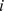 and all the other healthy breast tissues from the cohort.

Another way to see this phenomenon is to assess statistically, within each cohort, how the methylation distances between matched pairs differ from the methylation distances between unmatched pairs. Figure 3 displays the distributions of methylation distances for different sets of sample pairs compared to the distance between matched sample pairs. We also display in Figure 4 the boxplot of methylation distances by groups. Real matched pairs between a PT and its corresponding metastasis or recurrence are significantly closer in terms of global methylation than a random pair of samples taken from two different individuals, both in the PT/AM cohort (P-value = 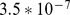) and in the PT/LR cohort (P-value = 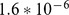). This is however not true in the PT/CL cohort, where we detect no differences between correctly and randomly matched pairs (P-value = 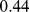). In addition, we calculated the distribution of distances between the CL tumors. We performed the same analysis between the PT tumors. We observed that the distribution were not significantly different (P-value = 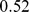), as expected. This is in agreement with the assumption we made that CL tumors could be considered as new primary tumors. Finally, we also compared the distribution of distances between the healthy breast tissue 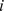 and all the other healthy breast tissues from the cohort to assess the heterogeneity between normal breast tissues.

### Clonality detection based on methylation profiles

The above results suggests that methylation profiles tend to be conserved during clonal expansion (such as samples in the PT/AM cohort), but strongly differ between unrelated tumors in a given person (such as samples in the PT/CL cohort). Moreover, methylation seems to be a stable mechanism in normal tissues compared to cancerous ones. It is therefore tempting to use methylation distance as a tool to discriminate true recurrences from new tumors in ambiguous cases, that is, for samples in the PT/LR cohort.

**Figure 5:**
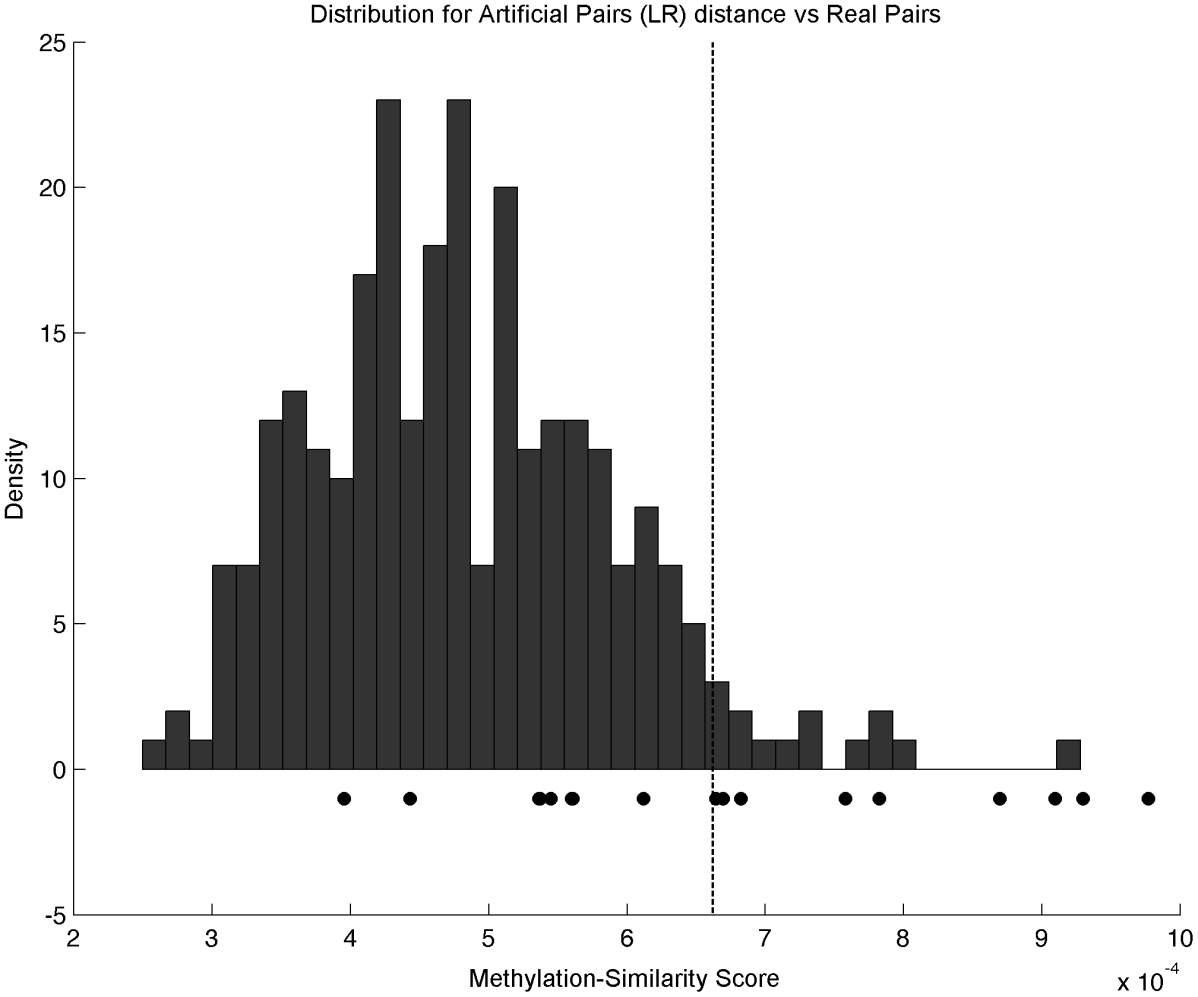
Histogram of the distribution of methylome-similarity score (MS) between unrelated PT/LR pairs. MS score for matched pairs is represented by circles. The vertical dashed line corresponds to the 95% quantile of the distribution of the MS scores for the unrelated pairs, used as a threshold to define clonal pairs (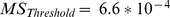).

9 out of 17 PT/LR pairs (52%) have a MS score higher than the threshold given by the 95% percentile of the MS score between unrelated pairs (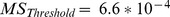) as shown in Figure 5; they are therefore considered as clonal pairs from the methylation point of view. The remaining 8 pairs are considered as non-clonal, meaning that the LR may correspond to a new primary tumor. Figure S2 shows how related pairs are similar compared to unrelated pairs for the PT/AM (Panel A) and PT/CL (Panel B) groups.

**Figure 6:**
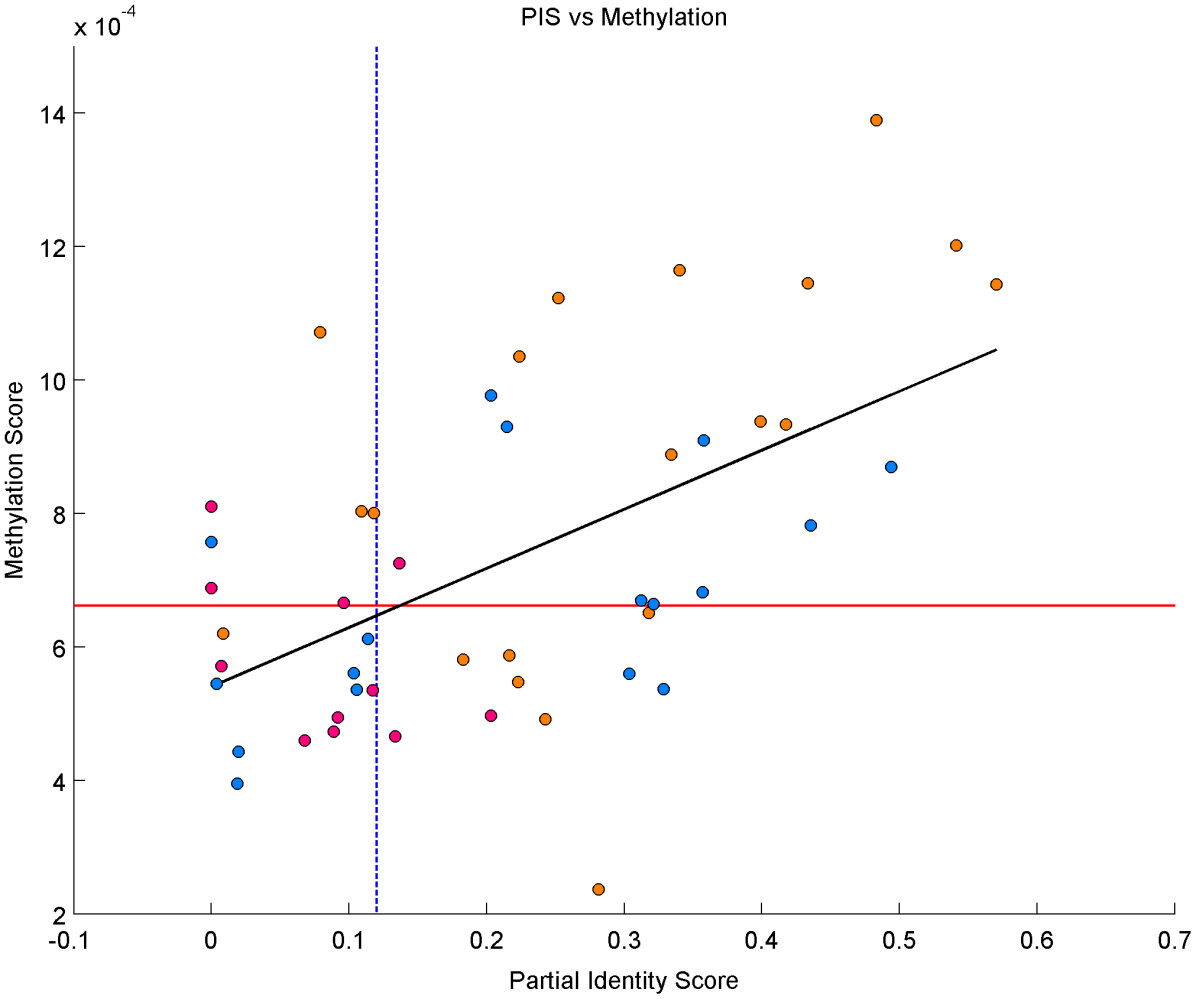
Correlation between methylation and copy-number scores. The horizontal red line (resp. vertical dashed blue line) corresponds to the 95% quantile of the distribution of the methylation-scores (resp. partial identity scores) for the unrelated pairs: 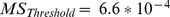 (resp. 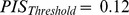). PT/AM (resp. PT/LR, resp. PT/CL) pairs are colored in yellow (resp. blue, resp. pink). The black line corresponds to the linear regression between methylation and copy-number scores for all the datasets.

Comparison between the methylation-based similarity measure MS score with the partial identity score (PIS), a copy-number based similarity measure developed by [16] show a good correlation overall (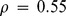, P-value = 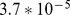, see Figure 6). Table 5 gives a comparison of the outcomes given by methylation-based, copy-number based and clinical-based classification of LR as TR or NP. The methylation-based classification method agreed with the copy-number based PIS classification method on 14 out of 17 pairs (concordance = 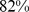, P-value = 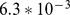) and agreed with the clinical-based classification on 14 out of 17 pairs (concordance = 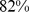, P-value = 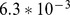).

**Table 5:** Comparison of classification methods for clonality between pairs in the PT/LR cohort.

Panel A								
Scores					Classification			
Pair	Cor	PIS	MS	Time	PIS	MS	Clinical	Divergence
			(×10^−4^)	(Years)				
1	1	0.019	4.42	6.5	NP	NP	NP	
2	3	0.435	7.82	3.2	TR	TR	TR	
3	11	0.018	3.95	6.4	NP	NP	NP	
4	16	0.303	5.59	3.8	TR	NP	NP	PIS
5	12	0.113	6.11	3.4	NP	NP	NP	
6	13	0.214	9.29	4.6	TR	TR	TR	
7	15	0.105	5.36	3.2	NP	NP	TR	Clinical
8	2	0	7.57	5.2	NP	TR	NP	MS
9	4	0.203	9.76	3.5	TR	TR	TR	
10	14	0.321	6.64	2.4	TR	TR	TR	
11	18	0.003	5.44	2.2	NP	NP	NP	
12	20	0.103	5.60	1.4	NP	NP	NP	
13	21	0.356	6.82	4.2	TR	TR	TR	
14	23	0.328	5.37	0.9	TR	NP	TR	MS
15	24	0.312	6.69	1.4	TR	TR	TR	
16	25	0.357	9.09	2.7	TR	TR	TR	
17	26	0.493	8.69	2.0	TR	TR	TR	

**Cor** (Correspondence): correspondence number with the Bollet/Servant cohort. **scores**: scores obtained with partial identity (PIS) or methylation (MS). **Time**: time elapsed between diagnosis of the PT and diagnosis of the recurrence. **Classification**: classification of the recurrence based on copy number (PIS), methylation (MS) or clinical features (clinical). **Divergence**: which method deviated from the others.

Finally, the different classifications of LR as TR or NP were correlated with time-to-recurrence and metastasis-free survivals. The differences in time-to-recurrence for the two groups defined by methylation-based classification or the clinical and histological classification were not statistically significant (P-value = 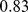 and P-value = 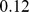). It was however significant using the partial identity score (P-value = 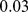) (Figure S3). This is interesting in the sense that one of the main criteria to distinguish TR and NP is the time-to-recurrence. Therefore, methylation-based classification is based on more information than time only.

**Figure 7:**
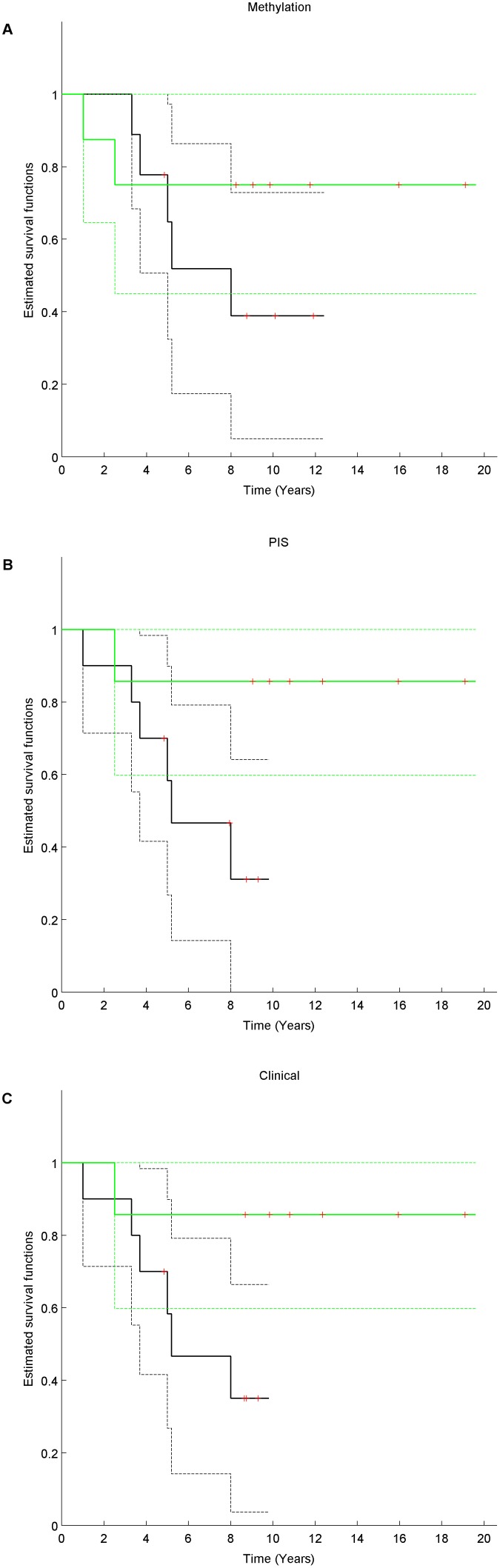
Kaplan-Meier estimates of the metastasis-free survival between TR and NP for the different classification methods. The full black (resp. green) line corresponds to the survival for samples classified as TR (resp. NP) and the corresponding dashed lines correspond to upper and lower 95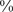 CI. The red crosses represent censored data. Panel A (resp. B, resp. C) represent the methylation-based (resp. copy-number based, resp. clinical based) classification.

The difference in metastasis-free survival of patients with TR and NP was not significant based on methylation (P-value = 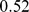, Hazard-Ratio = 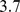, 5 year metastasis-free survival = 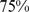 for NP), copy-number (P-value = 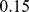, Hazard-Ratio = 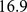, 5 year metastasis-free survival = 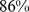 for NP) or clinical features (P-value = 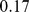, Hazard-Ratio = 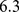, 5 year metastasis-free survival = 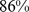 for NP) (Figure 7). Adjusting for age, grade and ER status did not yield more significant results except for copy-number based classification (P-value = 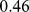, Table S6).

## Discussion

We studied alterations of methylation profiles from primary breast carcinomas and different types of recurrences, namely, axillary metastases, local recurrences and contralateral breast carcinomas. For this particular dataset, we observed significant methylation differences for 49 CpG probes, which characterizes the progression between a PT and its AM. Consistent with this result, a multivariate analysis with a linear SVM classifier using a small subset of probes perfectly distinguished PTs from AMs with a 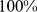 accuracy. Several significantly differentially methylated probes correspond to genes involved in cancer-related mechanisms such as cell death (*MCF2L*, *RASSF5*, *RASSF6*, *CASZ1*, *SLC22A18*, *IFI27*), tumorogenesis (*CTSZ*, *TP73*, *CTSK*, *PIK3R1*), *KLK11*, cell cycle (*PPM1G*, *RANBP5*, *VAMP8*) and cell differentiation (*SMAF1*, *PAX6*, *PAX8*). On the contrary, for the PT/LR and PT/CL sets, univariate analyzes were not able to find significantly differentially methylated probes. This absence of specific epigenetic alterations between the primary tumors and the local recurrences or the contralateral breast recurrences was confirmed by the poor performances of linear classifiers, unable to separate PT from LR nor PT from CL significantly better than random guesses. Nevertheless, the absence of methylation markers in the PT/LR and the PT/CL groups does not necessarily mean that the primary tumor and the recurrence are independent. We cannot rule out the possibility that the recurrence arises from a specific subclone which does not match the major subclone of the primary tumor. One could for example analyze the methylation profiles of several microdissections samples of the primary tumor to study potential heterogeneity.

The second part of the study focused on observing stability in methylation profiles. It is interesting to note that although PTs and AMs were significantly differentiable using a subset of probes, they also have overall very similar methylation profiles indicating that the tumors might actually be clones with specific alterations characteristic of the lymph node status. The subset of genes determined in the first part, if confirmed, could be associated with bad prognosis. On the other part, although the LRs and the CLs were not significantly different from their primary tumors, they tend to have overall different methylome profiles especially for the CLs. The overall different methylome profiles for the PT/CL set was expected since CLs are usually considered to be independent tumors.

The results above suggested to use global methylation analysis as a measure of clonality to tackle the subclonal populations in the local recurrences as proposed by Veronesi et al. [9]. A methylation-based classification was proposed to distinguish LRs as either true recurrences of the first PT or new PT [10]. A comparison with both clinical and copy-number based classifications on the same cohorts agreed on 14 out of 17 samples (82% concordance, P-value = 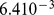) for both methods, although comparisons on larger cohorts are needed to assess the performance of methylation-based classification. Moreover, a good correlation between the methylation-based similarity score and the copy-number based similarity score seems to indicate a link between modifications at the genomic and epigenomic levels. Although the role of methylation in gene expression has thoroughly been studied [24]–[26], the relationship between methylation and copy-number still remains unclear. Houseman et al. [33] note that there is a negative bias of methylation when one or both alleles are lost but none in case of gains. Several other studies have reported correlation between the two mechanisms in different types of cells. Strong associations have been reported in urothelial carcinoma [34], head and neck squamous cell carcinomas [35], and mesothelioma [36]. Our study provides new evidence for association between methylation and copy-number on a global scale.

The discordances between the methylation-based classification method and the usual clinical method are discussed here for the samples 7, 8 and 14, although no actual method is a gold standard for classifying TR from NP. Sample 8 filled almost all the requirements for clinical classification as TR (location, receptor status) but failed in aggressiveness and type of tumor (PT was ductal type 2 and LR was lobular type 1). A decrease of aggressiveness of the recurrence could be explained by the use of neoadjuvant therapies. For the change of type, Fisher et al. showed that a mixing of ductal and lobular breast carcinoma was a possibility in 6% of the patients [37] which could explain the change in type. Sample 7 was classified as TR by clinical classification and as NP by both methylation and copy-number based classifications. This suggests some limitations to methods based only on clinical features.

An interesting question for clinical applications would have been to predict whether a primary tumor would relapse (either as AM, LR or CL) or not. However, the patient cohort used in this study does not allow to address this question. Indeed, one would require to compare the methylation profiles of patients who did not display any relapse (AM, LR and CL) to those of the current study.

## Materials and Methods

### Patients Selection

The patients were 49 years old or younger at diagnosis of the initial tumor; all patients were premenopausal; and had no previous history of cancer, except for one nonmelanoma skin cancer. The patients' PT was either ductal or lobular invasive breast carcinoma. However, both types of tumors did not display significantly differentially methylated probes and were thus all included in this study (min P-value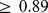).

Specimens from patients with primary breast cancers and breast cancer recurrences were selected from freshly frozen samples of the Institut Curie tissue bank according to the following criteria: all patients had been treated at the Institut Curie by breast-conserving surgery, including dissection of the axillary lymph nodes in most patients, followed by radiotherapy to the breast with or without a boost to the tumor bed (external beam radiotherapy or brachytherapy) and/or to the regional lymph node-bearing areas if indicated and, when required, systemic treatment as part of their initial management. Methylation profiles did not significantly differ depending on either ER, PR, HER2 and grade characteristics (min adjusted P-value = 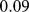).

To ensure that the data would be informative, genomic analyzes were restricted to tumors (primary and recurrences) in which at least 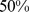 of cancer cells had been assessed by hematoxylin, eosin, and saffron staining of sections from snap-frozen samples. All the therapies were performed posterior to the biopsies of the primary tumors. Therefore, the studied methylation profiles are not modified by any potential effect of the treatments.

The 22 healthy breast tissues are taken from healthy women who underwent cosmetic plastic surgery at the Institut Curie. Part of the PT/AM cohort is identical to the cohort studied by Bollet et al. [16].

All experiments were performed retrospectively and in accordance with the French Bioethics Law 2004–800, the French National Institute of Cancer (INCa) Ethics Charter and after approval by the Institut Curie review board and ethics committee (Comit de Pilotage of the Groupe Sein). In the French legal context, our institutional review board waived the need for written informed consent from the participants. Moreover, women were informed of the research use of their tissues and did not declare any opposition for such researches. Data were analyzed anonymously.

### Methylation profiling

For each sample the methylation status at 27,578 positions in the genome was measured with the HumanMethylation27 BeadChip of Infinium technology [38] using the standard Illumina protocol. Quality control was assessed using in-built Illumina technology.

### Copy number based classification

The PIS score, based on copy number alterations similarities between the primary tumor and its recurrence, was retrieved from [16] for the same population.

### Clinical Classification

Histopathologic characteristics were reviewed by a single pathologist. The histological and biological properties of each sample was determined by subjecting tissue sections to immunohistochemical analysis for the estrogen receptor (clone 6F11, 1∶200 dilution; Novocastra, Newcastle Upon Tyne, England) and progesterone receptor (clone 1A6, 1: 200 dilution; Novocastra) antibodies. Tumors were considered to be positive for these receptors if at least 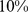 of the invasive tumor cells in a section showed nuclear staining [39], [40]. The HER2 analysis was performed using the standard ASCO guidelines [41]. In accordance with theories of the clonal evolution of tumor cell populations, LR were clinically defined as TR if they had the same histologic subtype (ductal or lobular) and a similar or increased growth rate, similar estradiol, progesterone and HER2 receptor statuses, and similar or decreased differentiation as the initial tumor [10]. TR also had to share with their PT the same breast quadrant. Thus, new PT were clinically defined as such when the LR had occurred in a different location, had a distinct histologic type, or had less aggressiveness features (lower grade, presence of hormonal receptors) than the initial tumor.

### Data analysis

A spatial normalization process was applied to all profiles [42]. Among the 27,578 probes measured on each sample, 5 probes were removed due to missing values for some individuals, and all subsequent analysis was performed on the 27,573 remaining probes.

Differentially methylated probes between PT and their matched AM, LR and CL are obtained using two-sided paired and unpaired Wilcoxon tests, correcting the p-values for multiple testing with the methods of Benjamini and Hochberg [43]. Multivariate analysis was performed using a linear support vector machine (SVM) multidimensional classifier on either the complete methylation profile or after dimensional reduction by considering only the most significant probes based on the Wilcoxon test. A p-value was calculated to assess the significance of the predictor accuracy compared to a predictor that would predict classes randomly. Unsupervised classifications were performed with complete linkage agglomerative clustering using the MATLAB bioinformatics toolbox, while the support vector machine implemented in LIBSVM [44] was computed with a linear kernel and nested leave-one-out cross validation for parameter selection for supervised classification.

The similarity between two copy number profiles is assessed with the partial identity score (PIS) as defined by Bollet et al. [16], which is based on the quantity of shared breakpoints between the two profiles and their frequencies. Following [16], a recurrence from a matched PT/LR pair was considered TR based on copy numbers when the PIS between the PT and LR profiles was above the 95% quantile of the empirical PIS distribution between unrelated sample pairs. Similarly, a Methylation-Similarity score (MS) is defined based on the methylation profiles of a PT and its matched LR as the inverse of the Manhattan distance between their methylation profiles considered as 27,573-dimensional vectors. LR are then classified as TR of its matched PT when the MS score is above the 95% quantile of the empirical MS distribution between unrelated pairs. As a baseline, these results were compared to the Manhattan distance between unrelated normal breast tissues.

Metastasis-free survival was estimated by the Kaplan-Meier Method [45] and compared between the group of patients who were diagnosed as TR and the group diagnosed as NP using the log-rank test. The confidence interval of the hazard ratio was obtained using a semi-parametric Cox model [46]. Computation was done using MATLAB packages Logrank [47] and KMPlot [48].

## Supporting Information

Figure S1
**Quantile-quantile plot of the Wilcoxon test statistics for each groups.** Plot of the data quantiles (black dots) against normal theoretical quantiles. The red line is 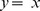.(TIF)

Figure S2 **Histograms of the distribution of Methylome-Similarity score (MS) between unrelated PT/AM and PT/CL pairs.** MS score for matched pairs is represented by crosses for the PT/AM pairs (Panel A) and by stars for the PT/CL pairs (Panel B). The vertical dashed line corresponds to the 95% quantile of the distribution of the MS scores for the unrelated pairs.
(TIF)

Figure S3 **Correlation between time to recurrence and classification of the recurrence.** Boxplots of time between the primary tumor and the local recurrence depending on the classification as true recurrence (TR) or new primary tumor (NP) according to the methylation-based, copy-number based (PIS) and clinical based classification.
(TIF)

Table S1 **Complete PT/LR Clinical and histological features.** **Cor** (Correspondence): correspondence number with the Bollet/Servant cohort from [16], **Type**: histological type of the tumor (D =  ductal, L =  lobular), **Grade**: Aggressiveness of the tumor (1 to 3), **ER**: percentage of estrogen receptors, **PR**: percentage of progesterone receptors present, **HER2**: presence of HER2 receptors, **Loc** (Location): 1 if the recurrence was located less than 4cm from the PT.
(TIF)

Table S2 **Complete PT/CL Clinical and histological features.** **Type**: histological type of the tumor (D =  ductal, L =  lobular, Med = Medullary, Meta = Metaplasic), **Grade**: Aggressiveness of the tumor (1 to 3), **ER**: percentage of estrogen receptors present, **PR**: percentage of progesterone receptors present, **HER2**: presence of HER2 receptors.
(TIF)

Table S3 **Complete PT/AM Clinical and histological features.** **Age**: Age of the patient at diagnosis of the primary tumor in years, **Type**: histological type of the tumor (D =  ductal, L =  lobular, Meta = Metaplasia), **Grade**: Aggressiveness of the tumor (1 to 3), **ER**: percentage of estrogen receptors present, **PR**: percentage of progesterone receptor present, **HER2**: presence of HER2 receptors.
(TIF)

Table S4 **Top 50 CpG loci between PT and LR samples.** **CpG**: CpG probe name. **Gene**: Associated gene. **Pvalue**: FDR corrected p-value. **Methylation Variation**: Mean variation of methylation from the primary tumor to the local recurrence.
(TIF)

Table S5 **Top 50 probes between PT and CL samples.** **CpG**: CpG probe name. **Gene**: Associated gene. **Pvalue**: FDR corrected p-value. **Methylation Variation**: Mean variation of methylation from the primary tumor to the contralateral recurrence.
(TIF)

Table S6 **Predictive impact of the classification methods on survival in breast cancer.** **Variables**: variable considered for predictive impact adjusted for the other variables present in the table. **Coef**: Associated coefficient in the Cox regression. **lower/upper .95**: lower and upper 95% confidence interval. **Pvalue**: P-value associated with the predictive impact on survival.
(TIF)
