# Supplementary figures and images for: Epigenomic Alterations in Breast Carcinoma from Primary Tumor to Locoregional Recurrences
Source: PLoS One. 2014 Aug 6;9(8):e103986. doi: 10.1371/journal.pone.0103986 (PMC4123987; doi:10.1371/journal.pone.0103986)

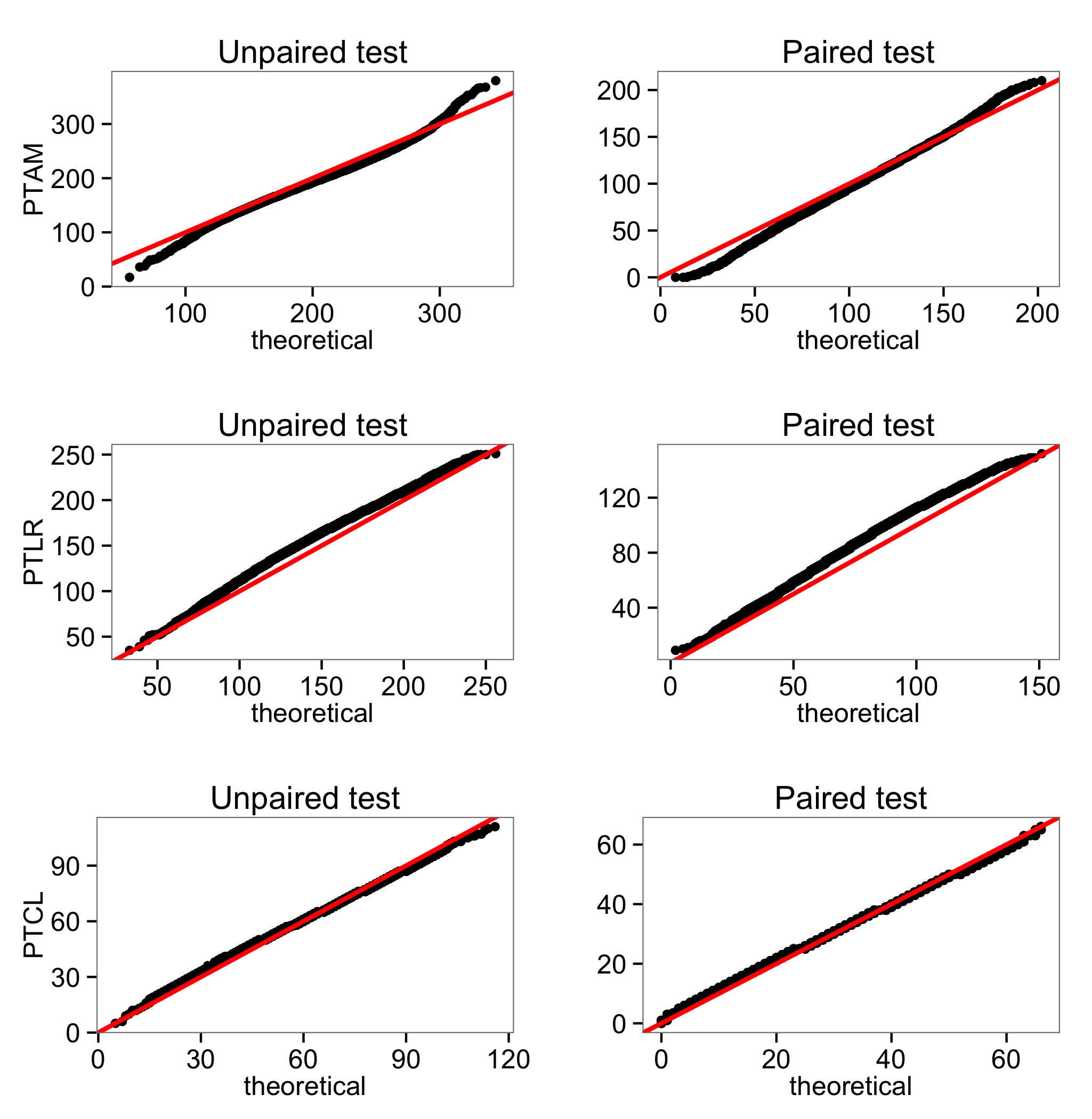

Supplement: Figure S1 — Quantile-quantile plot of the Wilcoxon test statistics for each groups. Plot of the data quantiles (black dots) against normal theoretical quantiles. The red line is . (TIF) [file pone.0103986.s001.tif]

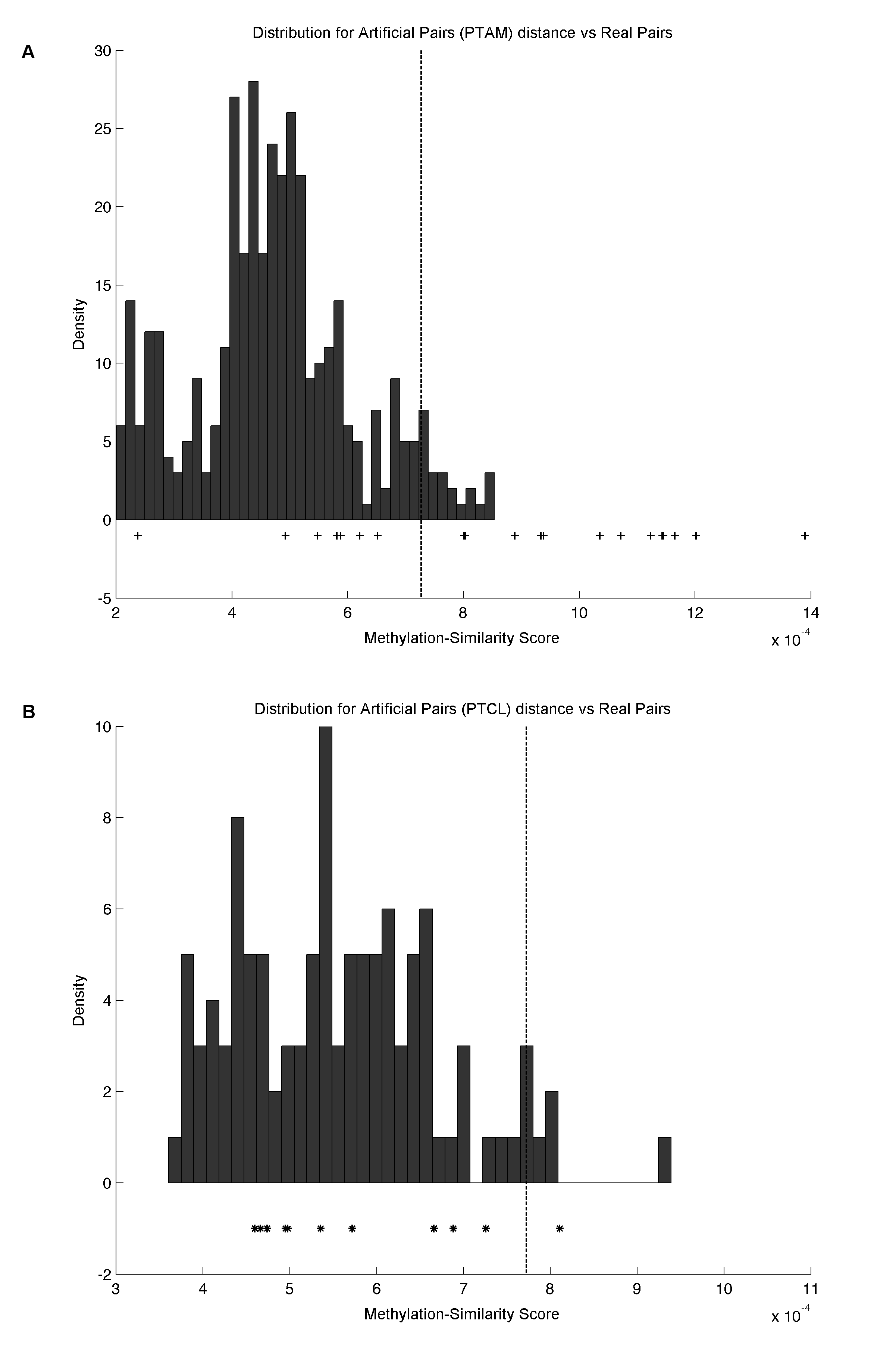

Supplement: Figure S2 — Histograms of the distribution of Methylome-Similarity score (MS) between unrelated PT/AM and PT/CL pairs. MS score for matched pairs is represented by crosses for the PT/AM pairs (Panel A) and by stars for the PT/CL pairs (Panel B). The vertical dashed line corresponds to the 95% quantile of the distribution of the MS scores for the unrelated pairs. (TIF) [file pone.0103986.s002.tif]

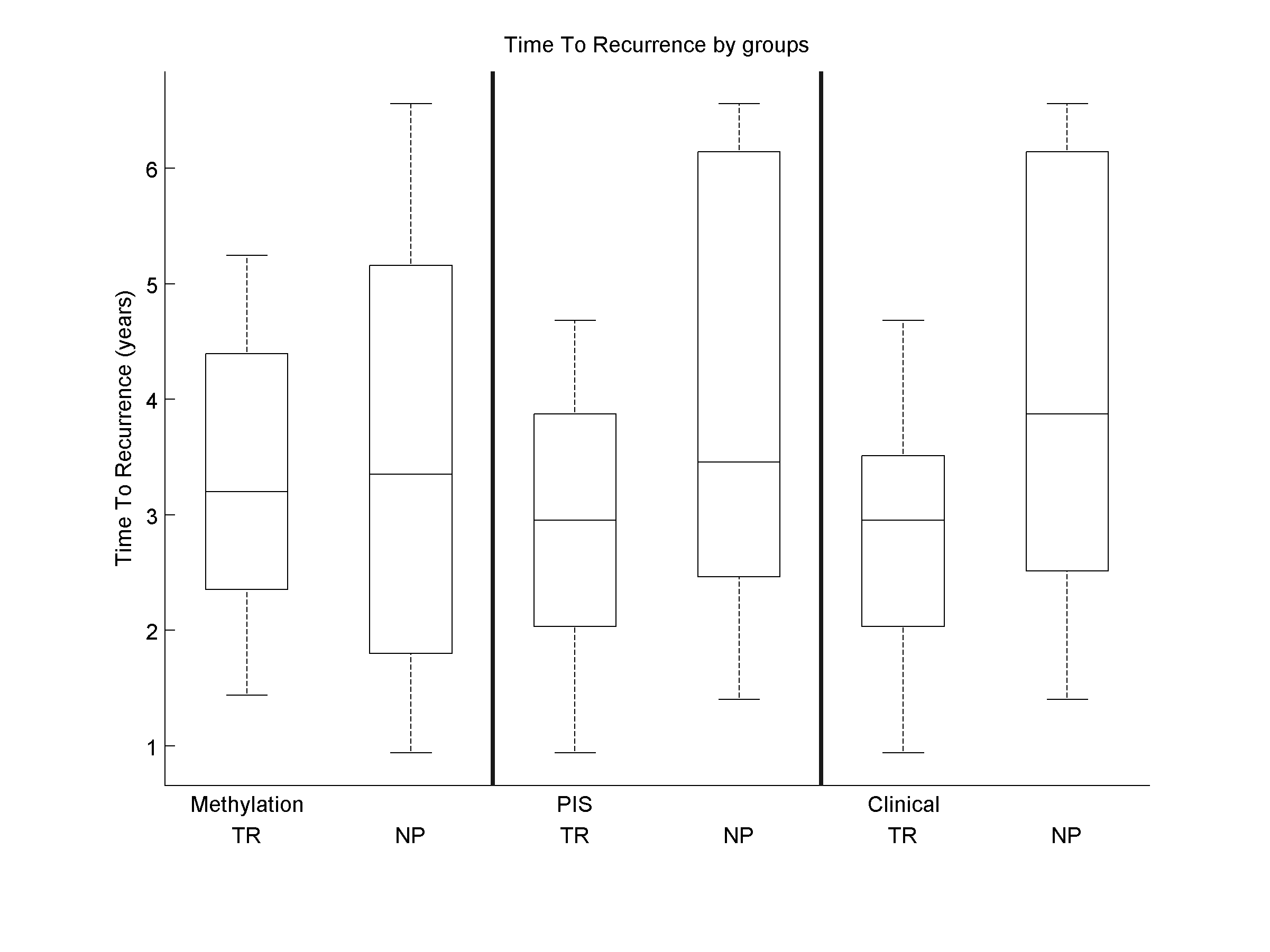

Supplement: Figure S3 — Correlation between time to recurrence and classification of the recurrence. Boxplots of time between the primary tumor and the local recurrence depending on the classification as true recurrence (TR) or new primary tumor (NP) according to the methylation-based, copy-number based (PIS) and clinical based classification. (TIF) [file pone.0103986.s003.tif]

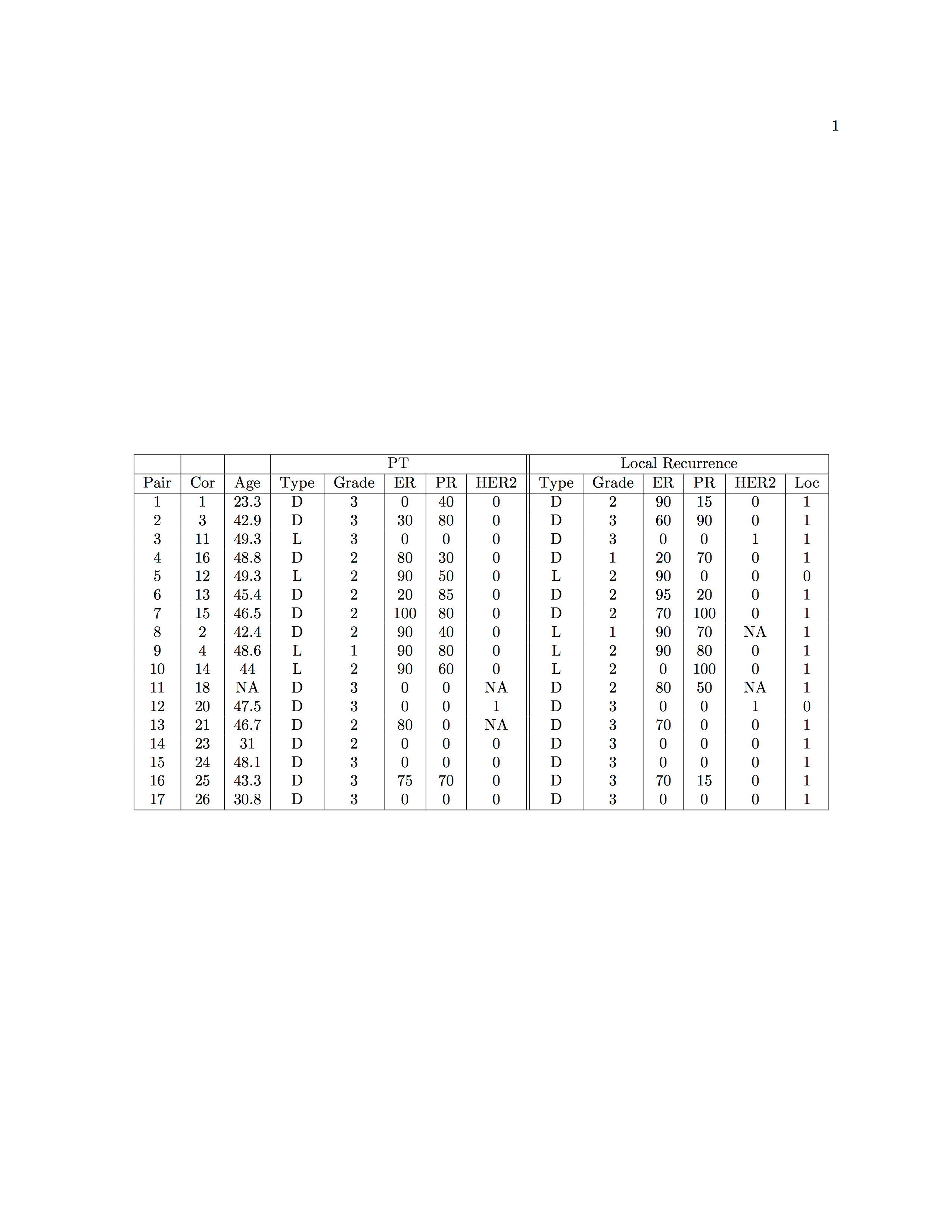

Supplement: Table S1 — Complete PT/LR Clinical and histological features. Cor (Correspondence): correspondence number with the Bollet/Servant cohort from [16], Type: histological type of the tumor (D = ductal, L = lobular), Grade: Aggressiveness of the tumor (1 to 3), ER: percentage of estrogen receptors, PR: percentage of progesterone receptors present, HER2: presence of HER2 receptors, Loc (Location): 1 if the recurrence was located less than 4cm from the PT. (TIF) [file pone.0103986.s004.tif]

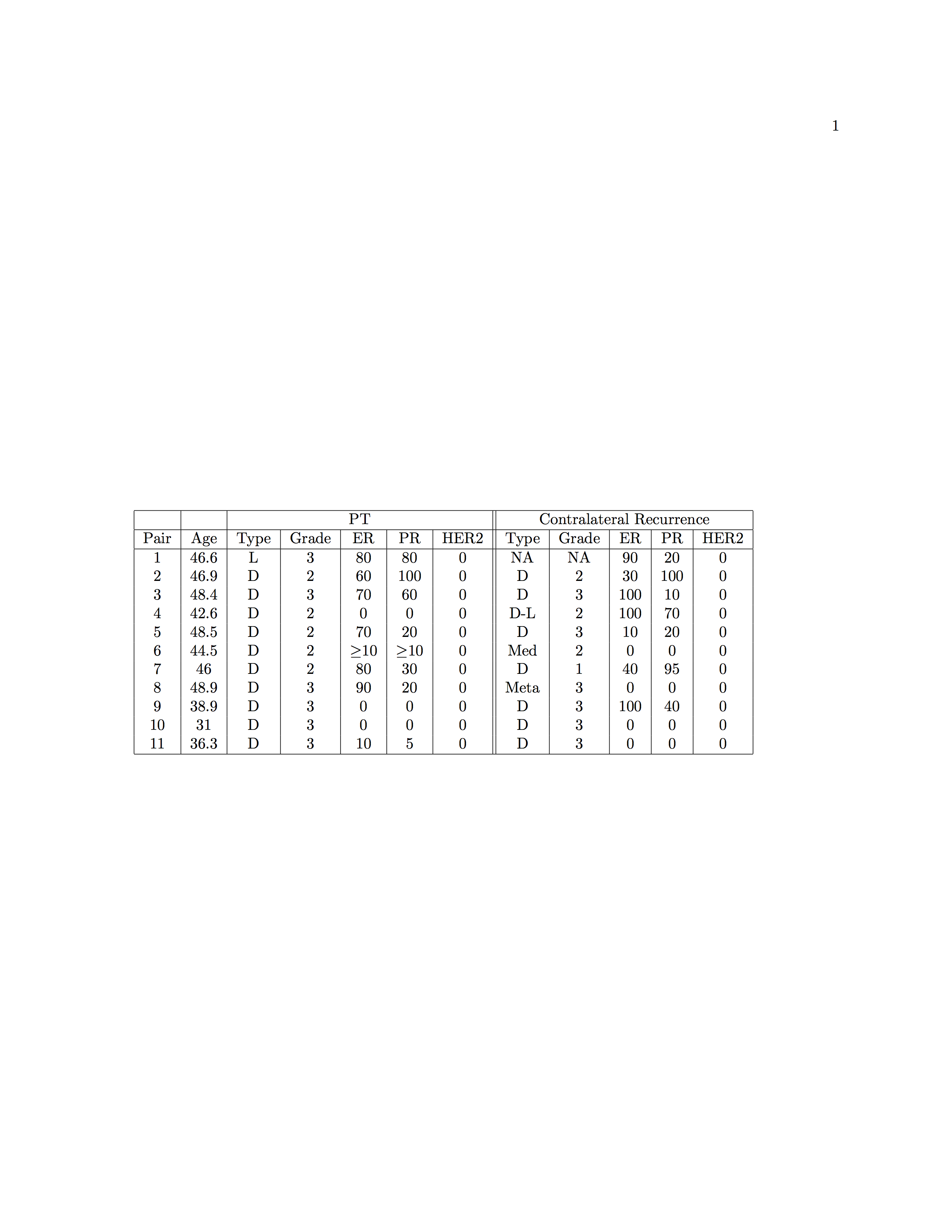

Supplement: Table S2 — Complete PT/CL Clinical and histological features. Type: histological type of the tumor (D = ductal, L = lobular, Med = Medullary, Meta = Metaplasic), Grade: Aggressiveness of the tumor (1 to 3), ER: percentage of estrogen receptors present, PR: percentage of progesterone receptors present, HER2: presence of HER2 receptors. (TIF) [file pone.0103986.s005.tif]

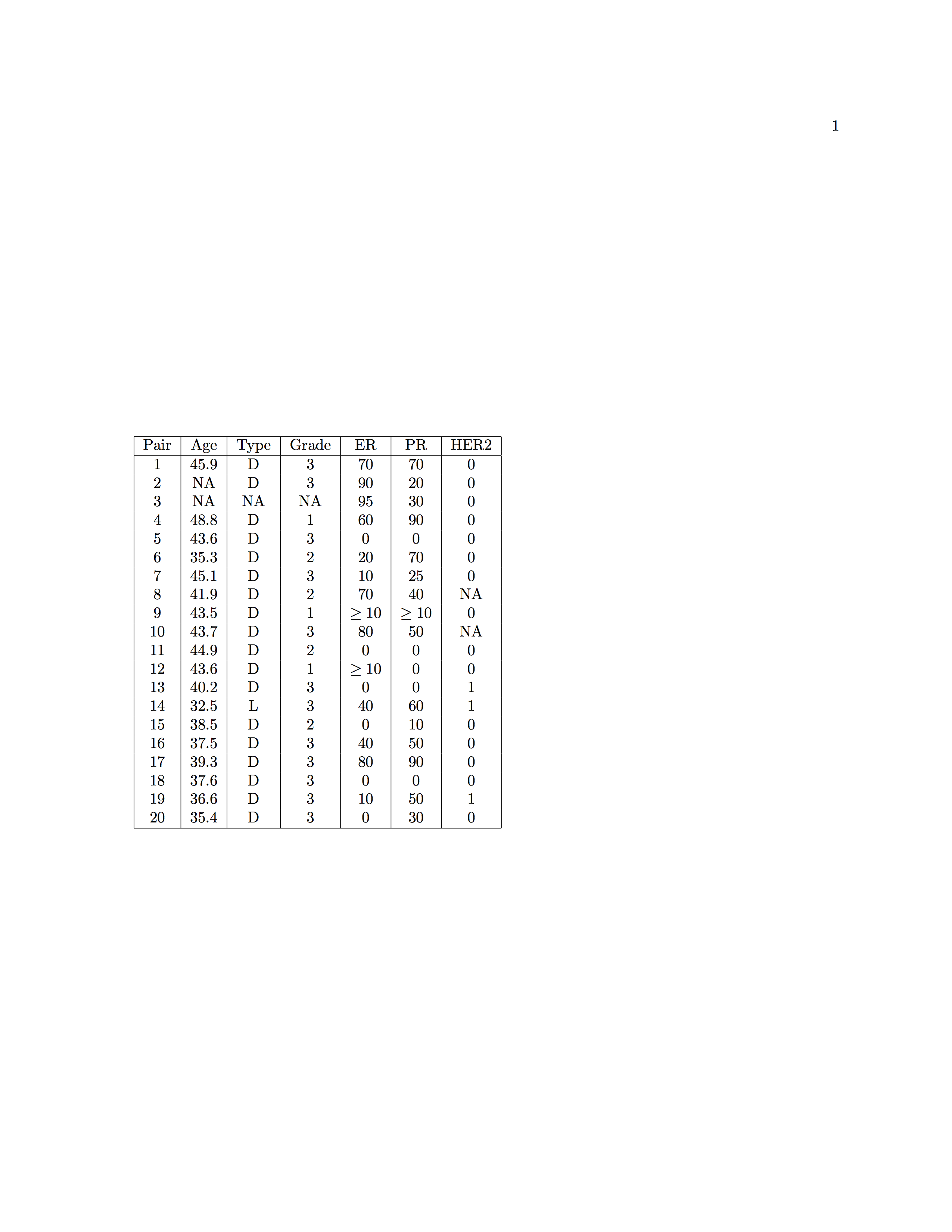

Supplement: Table S3 — Complete PT/AM Clinical and histological features. Age: Age of the patient at diagnosis of the primary tumor in years, Type: histological type of the tumor (D = ductal, L = lobular, Meta = Metaplasia), Grade: Aggressiveness of the tumor (1 to 3), ER: percentage of estrogen receptors present, PR: percentage of progesterone receptor present, HER2: presence of HER2 receptors. (TIF) [file pone.0103986.s006.tif]

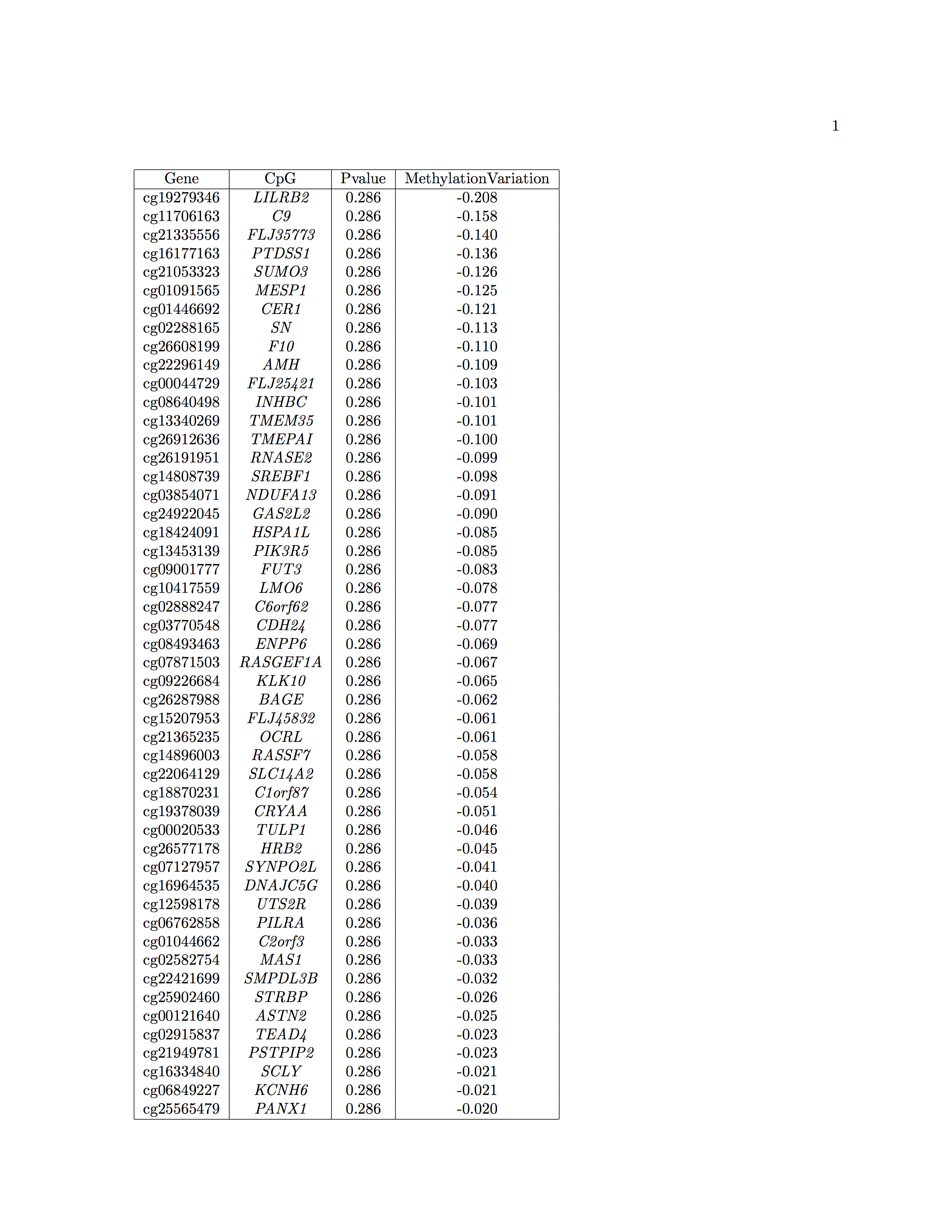

Supplement: Table S4 — Top 50 CpG loci between PT and LR samples. CpG: CpG probe name. Gene: Associated gene. Pvalue: FDR corrected p-value. Methylation Variation: Mean variation of methylation from the primary tumor to the local recurrence. (TIF) [file pone.0103986.s007.tif]

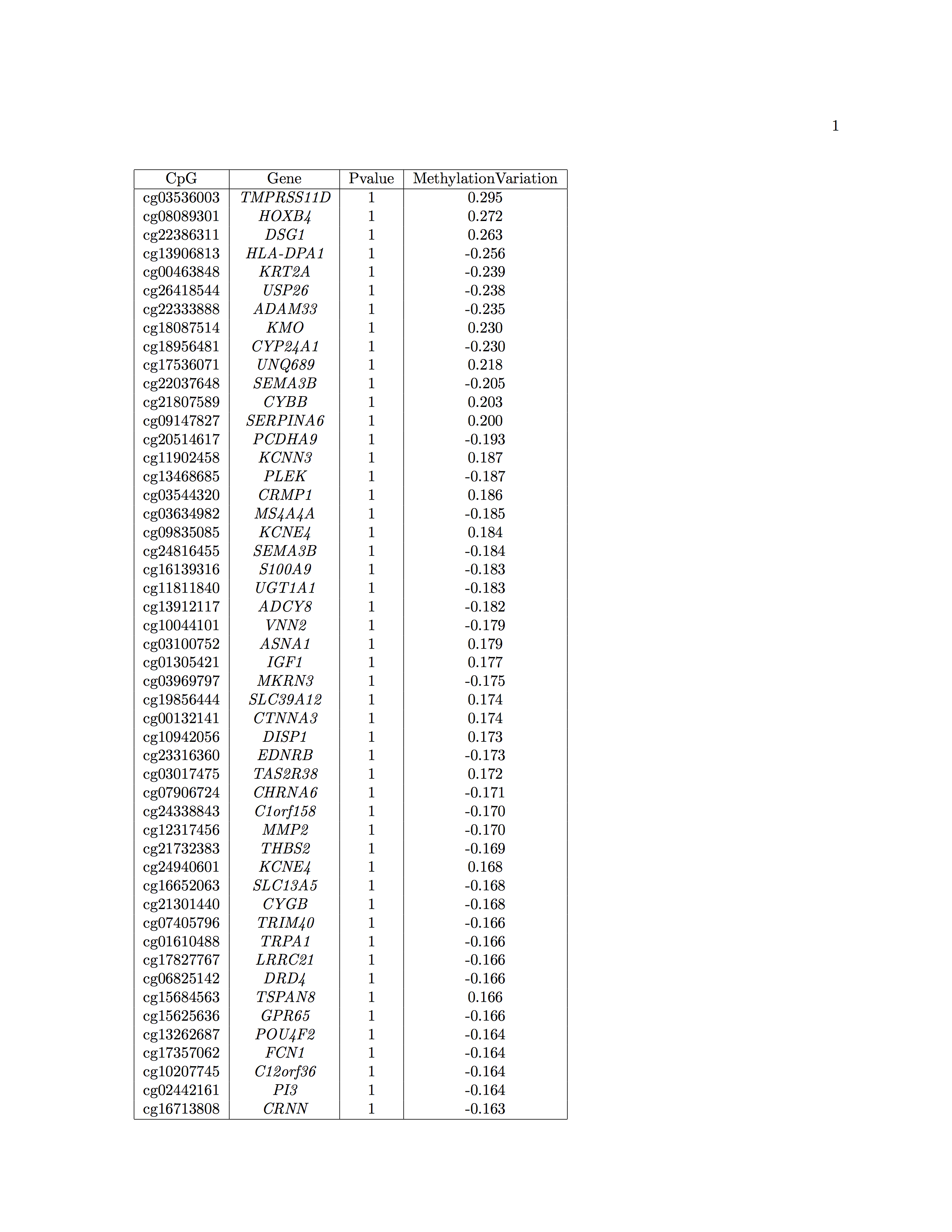

Supplement: Table S5 — Top 50 probes between PT and CL samples. CpG: CpG probe name. Gene: Associated gene. Pvalue: FDR corrected p-value. Methylation Variation: Mean variation of methylation from the primary tumor to the contralateral recurrence. (TIF) [file pone.0103986.s008.tif]

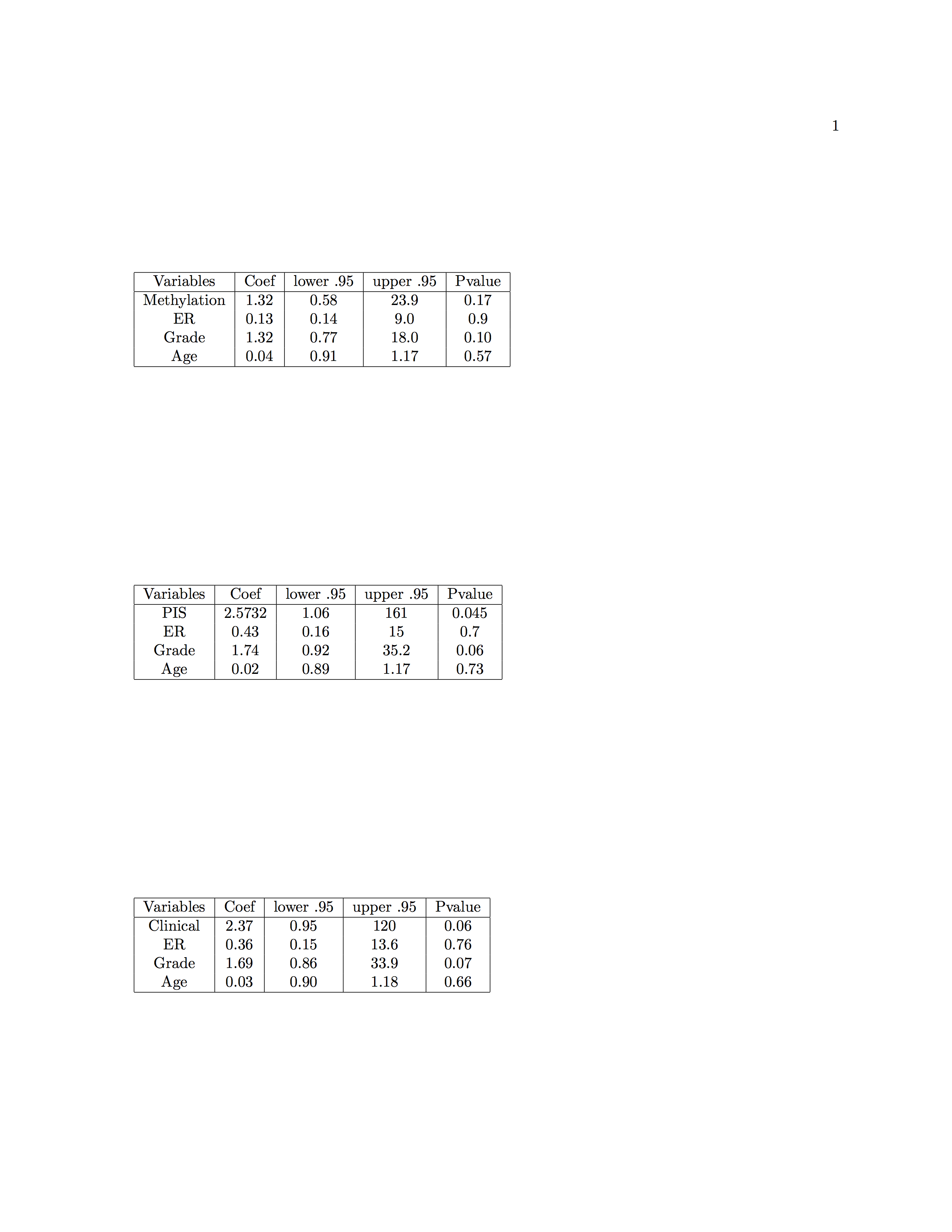

Supplement: Table S6 — Predictive impact of the classification methods on survival in breast cancer. Variables: variable considered for predictive impact adjusted for the other variables present in the table. Coef: Associated coefficient in the Cox regression. lower/upper .95: lower and upper 95% confidence interval. Pvalue: P-value associated with the predictive impact on survival. (TIF) [file pone.0103986.s009.tif]
